# Clinical characteristics and risk factors of coronary artery lesions in chinese pediatric Takayasu arteritis patients: a retrospective study

**DOI:** 10.1186/s12969-023-00820-z

**Published:** 2023-04-28

**Authors:** Yingjie Xu, Lingfeng Luo, Gaixiu Su, Jia Zhu, Min Kang, Dan Zhang, Jianming Lai, Xiaohui Li

**Affiliations:** 1 Capital Institute of Pediatrics-Peking University Teaching Hospital, Beijing, China; 2 Department of Rheumatology, Capital Institute of Pediatrics, Beijing, China; 3 Department of Cardiology, Capital Institute of Pediatrics, Beijing, China; 4 Department of Occupational and Environmental Health, School of Public Health, Suzhou Medical College, Soochow University, Suzhou, China

**Keywords:** Takayasu arteritis, Pediatric, Coronary involvement, Risk factors, Coronary aneurysmal dilation

## Abstract

**Backgroud:**

To summarize the clinical characteristics and identify the risk factors for pediatric Takayasu arteritis (TAK) with coronary artery lesions (CALs).

**Methods:**

Clinical data of pediatric TAK patients in our center were retrospectively assessed. Independent risk factors for CALs were identified using multivariate logistic regression analysis. Survival analysis was used to compare differences in survival rates between the groups.

**Results:**

Among the 66 pediatric TAK cases, the incidence of accompanying CALs was 39.4%. In the CAL group, 19 (73.1%) cases started within 36 months. None of the patients had symptoms of angina or ischemia on electrocardiogram (ECG), the CALs were detected using coronary ultrasound. The CALs most commonly were the left main and right coronary arteries. The lesions were mostly small or middle coronary artery aneurysms; some children may have giant coronary aneurysmal dilations, thrombosis and heart failure. The age of onset and symptom onset to diagnosis in TAK patients with CAL were lower than those in TAK patients without CAL(P < 0.005). TAK patients with CAL had significantly higher CRP,WBC, PLT,TNF-α and IL-2R levels (P < 0.05), lower HGB (P = 0.01), lower rate of renal artery stenosis (RAS) (P = 0.009). In multivariate logistic regression, the risk factors for pediatric TAK combined with CAL included the age of TAK onset (OR = 0.9835, 95% CI: 0.9710–0.9946, P = 0.006) and RAS (OR = 0.1901, 95% CI: 0.0386–0.7503, P = 0.03). In addition, there was no significant difference in survival rates between the two groups after regular treatment.

**Conclusion:**

This study showed that the occurrence of CAL in pediatric TAK patients has a relatively more rapid clinical course, and a stronger inflammatory state at the time of diagnosis. The earlier the age of TAK onset and without RAS are more likely to cause CAL.

**Supplementary Information:**

The online version contains supplementary material available at 10.1186/s12969-023-00820-z.

## Backgroud

Takayasu arteritis (TAK) refers to full-thickness arterial inflammation involving the aorta and its main branches that leads to arterial lumen stenosis or occlusion [1]. TAK complicated by coronary artery lesions (CAL) leads to myocardial infarction (MI), which causes TAK-associated death [2–4]. Although TAK is more common in adult females than in children and infants^[5.6]^, recent studies have reported that pediatric patients with TAK may have CALs ^[7.8]^. Wilson et al. [9] presented three adolescent females with TAK and MI who underwent balloon angioplasty, three-vessel coronary artery bypass grafting, and cardiac transplantation. When complicated by coronary abnormalities, the prognosis is generally poor because of the non-specificity of clinical manifestations with delayed diagnosis and difficulty of treatment. Therefore, it is important to further study and determine the clinical characteristics and risk factors of pediatric TAK complicated by CAL, and identify high-risk groups and provide effective intervention measures early to improve their prognosis.

It has been reported ^[10.11]^ that adults with TAK complicated with CALs were primarily related to the age of onset, disease duration, and atherosclerosis-related factors such as triglyceride (TG) and high-density lipoprotein cholesterol (HDL-C) levels. A recent study [12] compared the clinical characteristics of nine pediatric and 29 adult TAK patients with CAL. Children with TAK and CALs had higher disease activity than their adult counterparts on the first positive coronary assessment. The incidence of coronary aneurysmal dilation was higher in the pediatric group. To the best of our knowledge, this is the largest sample size study on the evaluation of CALs and the associated risk factors in pediatric TAK patients. However, the mean age at diagnosis of pediatric TA patients was 14.3 years old, which did not include children of all ages. The early research of our center found that children with TAK, especially infants and young children, have many blood vessels involved, severe systemic symptoms, poor prognosis, and some children have growth and development restrictions. In addition, infants and young children with TAK cannot express symptoms of hypertension such as dizziness and headache, so they are more likely to be misdiagnosed ^[13.14]^. In addition, it is possible that some of the underlying pathogenic mechanisms in pediatric TAK are different from those in adults [15–17]. Therefore, data for adults or adolescents cannot represent all pediatric TAK patients.

Thus, we conducted this study to summarize and analyze the clinical characteristics and risk factors of pediatric TAK combined with CAL, including infants, and to provide an evidence-based basis for the early screening of high-risk children with TAK.

## Materials and methods

This study was conducted in accordance with the Declaration of Helsinki and approved by the ethics committee of the Capital Institute of Pediatrics.

**Fig. 1:**
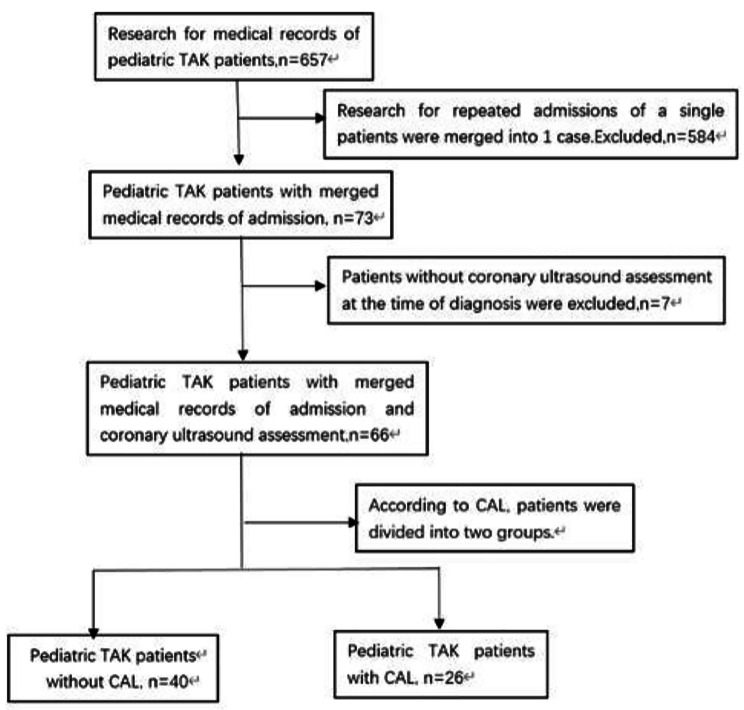
Consort flow diagram

***Patients.*** This retrospective cohort study included all pediatric patients with TAK who were admitted to the Children’s Hospital affiliated with the Capital Institute of Pediatrics between January 2009 and December 2021. A case search was initially conducted in the hospital information system using the International Classification of Diseases, 10th version code for TAK (M31.4). Two independent reviewers confirmed the diagnosis of TAK, and patients with other types of vasculitis, including Kawasaki disease (KD), were excluded. Pediatric TAK was diagnosed in patients with disease onset ≤ 18 years of age who met the European League Against Rheumatism (EULAR)/ Pediatric Rheumatology International Trials Organization (PRINTO)/ Pediatric Rheumatology European Society (PReS) classification criteria for TAK [18]. Patients with incomplete information or without coronary ultrasound evaluation at the time of diagnosis were excluded. According to CAL, the patients were divided into two groups: pediatric TAK patients with CAL (CAL group) and pediatric TAK patients without CAL (non-CAL group). The definition of CAL includes [19] coronary artery stenosis, obstruction, tumor-like expansion, fistula, and other lesions. Coronary artery dilatation was used to calculate the Z-value of the coronary artery based on sex, age, weight, and echocardiographic records, and was classified as: (1) No coronary artery dilation (< 2.0); (2) Coronary artery dilation (2.0~< 2.5); (3) Small coronary artery aneurysm (2.5~< 5.0); (4) Middle coronary artery aneurysm (5.0~< 10.0) and the absolute value of inner diameter < 8.0 mm; (5) Large and/or giant coronary aneurysms (≥ 10.0) or absolute diameter ≥ 8.0 mm. Except for non-dilated coronary arteries, these are collectively referred to as CALs. (Fig. 1)

***Data collection.*** The data included demographics, history, disease course, signs and symptoms, clinical classification, disease activity, laboratory tests, angiographic results, follow-up time, and outcomes. The disease duration was calculated from the first clinical manifestation that could be recalled and confirmed. Clinical classification of TAK based on the location of Lupi-Herrea vascular lesions, it can be divided into (1) brachiocephalic artery type involving the aortic arch and branches; (2) thoracoabdominal aortic type, involving the thoracic aorta, abdominal aorta, and branches; (3) extensive type, which has the clinical characteristics of both abovementioned types; and (4) pulmonary artery type, involving the pulmonary artery and any systemic artery. The disease activity criteria were adopted from the disease activity index proposed by Kerr et al. [20]. The score includes: (1) systemic symptoms, such as fever and skeletal muscle symptoms; (2) average erythrocyte sedimentation rate (AESR) increased rapidly; (3) vascular ischemia or inflammation, such as intermittent claudication, weakened or no pulse, vascular murmur andpain, and asymmetric blood pressure; (4) abnormal angiography. A score ≥ 2 points indicated disease activity. Laboratory tests included white blood cells (WBC), hemoglobin (HGB), platelet count (PLT), C-reactive protein (CRP), AESR, ferritin (FS), tumor necrosis factor-alpha (TNF-α), interleukin-6 (IL-6), IL-2 receptor (IL-2R), IL-8, total cholesterol, low-density lipoprotein cholesterol (LDL-C), HDL-C, TG, and immune function. Angiographic results included coronary, vascular B-flow, and intracranial Doppler ultrasonography, magnetic resonance angiography (MRA), and arterial computed tomography (CT). Arterial hypertension was defined as in-office blood pressure (systolic and/or diastolic) ≥ 95th percentile for individuals with matched age, sex, and height, as suggested in the guidelines ^[21.22]^.

***Statistical analysis.*** All data were entered into EpiData and statistically analyzed using SPSS software (version 20.0; IBM SPSS Inc., Chicago, USA). Continuous variables are expressed as mean ± standard deviation (mean ± SD) or median (interquartile range), and classified variables are expressed as frequencies and percentages [n (%)]. The chi-square test was used to compare groups of classified variables, and the skewness kurtosis test was used to determine whether continuous variables were normally distributed. An independent sample t-test was used to compare groups that met the normal distribution, and the nonparametric rank-sum test was used to compare groups with non-normal distribution. A multivariable logistic regression analysis was performed to identify risk factors associated with TAK, using variables with a significant intergroup difference (p < 0.05) between pediatric patients with and without CAL. Survival analysis was used to compare the difference in survival rates between groups. All tests were performed using a two-sided, and the level of statistical significance was set at P < 0.05.

## Results

### Clinical characteristics and coronary artery involvement in the CAL group (Table 1)

**Table 1:** Comparison of demographic, clinical characteristics, and laboratory findings between CAL group and non-CAL group

	Total N = 66	CAL group N = 26	non-CAL group N = 40	P values
Age of onset, months	85.2 (12.0-133.8)	12.0 [3.1–48.7]	115.6[82.1–146.0]	< 0.001
Gender				
female, n (%)	43 (65.2)	18 (61.2)	25 (62.5)	0.77
Symptom onset to diagnosis, day	20.0 (8.0-31.5)	11.5 [7.0–20.0]	28.0 [14.8–60.0]	0.01
Clinical manifestation, n (%)				
Fever	38 (57.6)	18 (69.2)	20 (50.0)	0.20
No pulse/weak pulse	18 (27.3)	7 (26.9)	11 (27.5)	1.00
Hypertension	30 (45.5)	8 (30.8)	22 (55.0)	0.09
Malaise	10 (15.2)	2 (7.7)	8 (20.0)	0.31
vascular murmur	14 (21.2)	4 (15.4)	10 (25.0)	0.53
Laboratory findings				
CRP, mg/L	41.5 [3.8-105.3]	64.5 [22.2-139.8]	25.0 [3.2–82.5]	0.04
AESR, mm/h	46.0 [12.5–89.5]	47.5 [18.2–86.0]	41.5 [11.8–95.0]	0.78
WBC, 10^9/L	10.3 [7.8–16.5]	13.9 [9.9–18.8]	8.7 [7.3–12.1]	0.002
HGB, g/L	106.7 ± 21.3	99.4 ± 19.8	111.4 ± 21.1	0.02
PLT, 10^9/L	401.0 [278.3-512.8]	481.5 [310.8-596.5]	346.5 [253.2-428.8]	0.01
FIB, g/L(2–4)	11.0 [9.0-17.7]	3.5[3.0-4.7]	4.9[2.9–4.6]	0.95
Na, mmol/L(135–145)	8.5 [4.7–20.0]	139.0[136.0-140.8]	139.9[136.0-141.0]	0.95
Albumin, g/l(35–55)	408.5 [341.5–659.0]	37.1[32.5–41.1]	39.5[36.0-43.1]	0.11
TNF-a, pg/mL	27.0 [15.0–37.0]	14.4 [10.6–18.6]	10.0 [7.9–16.0]	0.01
IL-6, pg/mL	157.0 [89.0-292.3]	12.1 [6.1–26.0]	7.7 [4.4–15.8]	0.06
IL-2R, pg/mL	3.0 [3.0–3.0]	527.0 [365.5-1547.2]	380.0 [292.2-566.5]	0.03
IL-8, pg/mL	27.0 [15.0–37.0]	26.0 [15.0–37.0]	27.5 [15.8–40.5]	0.71
FS, ng/mL	157.0 [89.0-292.3]	195.5 [94.0-385.5]	123.0 [87.2-238.2]	0.16
NIH score,(mean ± SD)	3.0 [3.0–3.0]	3.0 [2.0–3.0]	3.0 [3.0–3.0]	0.29
Numano classification, n (%)				0.60
1	24(36.4)	11 (42.3)	13 (32.5)	
2	9(13.6)	4 (15.4)	5 (12.5)	
3	33(50.0)	11 (42.3)	22 (55.0)	
4	0	0	0	
Carotid artery stenosis(CAS), n (%)	8 (12.1)	3 (11.5)	5 (12.5)	1.00
Aorticinsufficiency, n (%)	1(1.5)	0 (0.0)	1 (2.5)	1.00
Aortectasia, n (%)	1(1.5)	0 (0.0)	1 (2.5)	1.00
Thoracic aortic stenosis (TAS), n (%)	9 (13.6)	3 (11.5)	6 (15.0)	0.97
Abdominal aortic stenosis (AAS), n (%)	9 (13.6)	2 (7.7)	7 (17.5)	0.44
Renal artery stenosis (RAS), n (%)	24 (36.4)	4 (15.4)	20 (50.0)	0.009
Renal hypoperfusion, n (%)	6 (9.1)	1 (3.8)	5 (12.5)	0.45

CRP, C-reactive protein; AESR, average erythrocyte sedimentation rate; WBC, white blood cell; HGB, hemoglobin; PLT, platelet; FIB, fibrinogen; TNF-α, tumor necrosis factor-alpha; IL-6, interleukin-6; IL-2R, interleukin-2 receptor; IL-8, interleukin-8; FS, ferritin; NIH, National Institutes of Health

Sixty-six pediatric TAK patients were selected. There were 26 (39.4%) cases in the CAL group; 19 (73.1%) cases started within 36 months, of which two (7.7%) started at one month. Eighteen (69.2%) patients had a fever, eight (30.8%) had hypertension, and eight (30.8%) had rash. 12 (46.2%) patients were misdiagnosed with atypical KD due to CAL. These patients were under three years of age and did not meet the diagnosis of typical KD. Among them, nine children received intravenous immunoglobulin (IVIG) and oral high-dose aspirin (ASP), but the inflammatory reaction was not controlled. The temperature of the three patients was normal after IVIG combined with oral prednisone (PRE), but fever recurred when PRE was stopped.

**Fig. 2:**
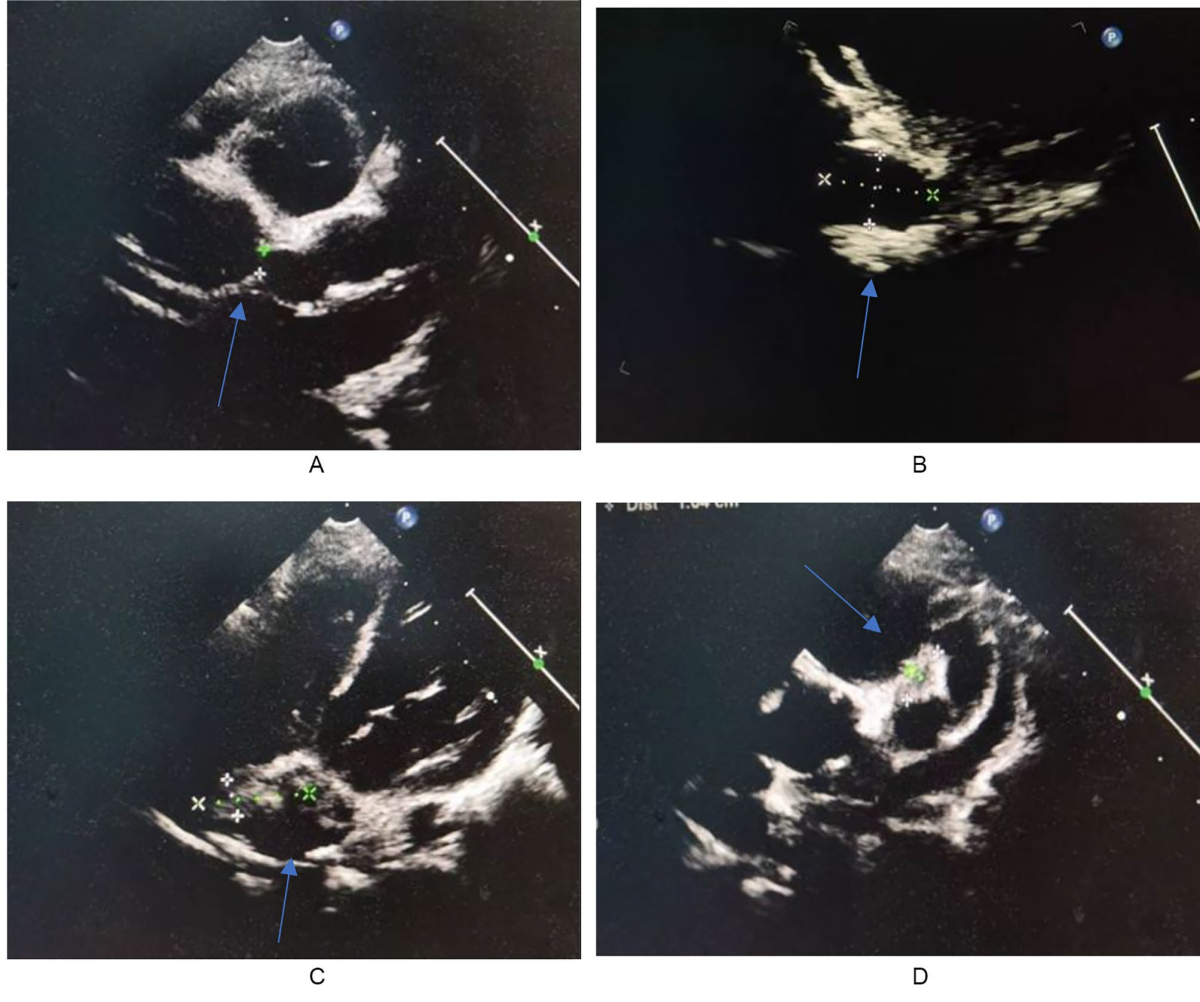
**A** Ultrasound showed aneurysmal dilation of the left main coronary artery in Patient(P) 8 (arrow). **B** Ultrasound showed aneurysmal dilation of the left main coronary artery in P9 (arrow). **C** Ultrasound showed right coronary artery thrombosis in P8 (arrow) **D** Ultrasound showed thrombosis in the left anterior descending coronary artery in P8 (arrow)

None of the patients had symptoms of angina or ischemia on electrocardiogram (ECG), the CALs were detected using coronary ultrasound. They were all dilated, without stenosis, occlusion and fistula. The most frequently affected coronary arteries were left main coronary artery (LMCA), right coronary artery (RCA), and left circumflex artery (CX), with the affected rates of 84.6%, 73.1%, 65.4% respectively. The rate of single, two, three and four vessel disease were 30.8%, 34.6%, 3.8% and 30.7% respectively. The median Z values of LMCA, LAD, CX and RCA were 5.3[3.1–6.5], 3.6[3.2–5.2], 3.0[2.8–3.8], 5.2[2.8–6.6] respectively, indicating that most CALs were small or middle coronary artery aneurysms. Five giant coronary aneurysmal dilations were formed with a maximum Z-value of 15.37. One case showed beaded changes, and two patients showed mural thrombosis (Fig. 2). Cardiac findings showed heart failure, pericardial effusion, and pulmonary hypertension in four, one, and two patients, respectively.

All 26 patients were treated with corticosteroids precombined with infliximab (IFX) and methotrexate (MTX), tocilizumab (TCZ) and MTX, and cyclophosphamide (CP) in 17 (65.4%), two (7.7%), and five (19.2%) cases, respectively. All the patients received ASP and/or warfarin as anticoagulant therapy. Four patients were administered anti-heart failure treatment simultaneously, and two patients received bosentan orally to reduce pulmonary artery pressure. The median follow-up time was four years, and 24 patients were stable. Nine patients with coronary artery dilatation recovered in > 1 year, and 14 regressed in more than 0.5 years. None of the patients developed MI or angina. Two patients died of irregular treatment during follow-up .

### Comparison of clinical characteristics and laboratory findings between pediatric TAK patients with and without CAL

Differences in clinical characteristics and laboratory findings between pediatric TAK patients with and without CAL are listed in Table 1. The age of onset and symptom onset to diagnosis in TAK patients with CAL were lower than those in TAK patients without CAL (P < 0.05). Laboratory findings showed that TAK patients with CAL had significantly higher CRP,WBC, PLT,TNF-α, and IL-2R levels than those in other patients (P < 0.05); HGB was significantly lower in patients with CAL than in other patients (P < 0.05). Among patients without CAL, the number of patients with renal artery stenosis (RAS) was significantly higher than that in the CAL group (P = 0.009). However, no statistical differences were found in the clinical manifestations, NIH score, disease activity, sex, fibrinogen (FIB), sodium (Na), and albumin. None of the patients had a history of smoking, drinking, diabetes, or a family history of hereditary hypercholesterolemia or hypertriglyceridemia. Serum total cholesterol, LDL-C, HDL-C, and TG levels were within normal ranges.

### The risk factors of pediatric TAK involving the coronary artery

We selected WBC, which showed the most significant difference between pediatric TAK with and without CAL, to represent the various inflammatory markers in the model. The final variables included in the multivariate logistic regression model were age of onset, symptom onset to diagnosis, WBC, TNF-a, and RAS. In multivariate logistic regression, the risk factors for pediatric TAK combined with CAL included the age of TAK onset (OR = 0.9835, 95%CI: 0.9710–0.9946, P = 0.006), and RAS (OR = 0.1901, 95%CI: 0.0386–0.7503, P = 0.03) (Table 2).

**Table 2:** Multivariate regression analysis of risk factors associated with coronary artery disease in pediatric TAK

	SE	χ2	OR	95% CI	P values
Age of onset	0.0060	7.7030	0.9835	0.9710–0.9946	0.006
Symptom onset to diagnosis	0.0043	0.0147	0.9995	0.9851–1.0060	0.90
WBC	0.0670	1.9210	1.0973	0.9681–1.2624	0.17
TNF-a	0.0164	0.0471	1.0036	0.9683–1.0384	0.83
RAS	0.7428	4.9950	0.1901	0.0386–0.7503	0.03

S.E., Standard error; χ2, chi-square test; OR, odds ratio; CI, confidence interval; WBC, white blood cell; TNF-α, tumor necrosis factor-alpha; RAS, renal artery stenosis

### Survival analysis of CAL and non-CAL groups

**Fig. 3:**
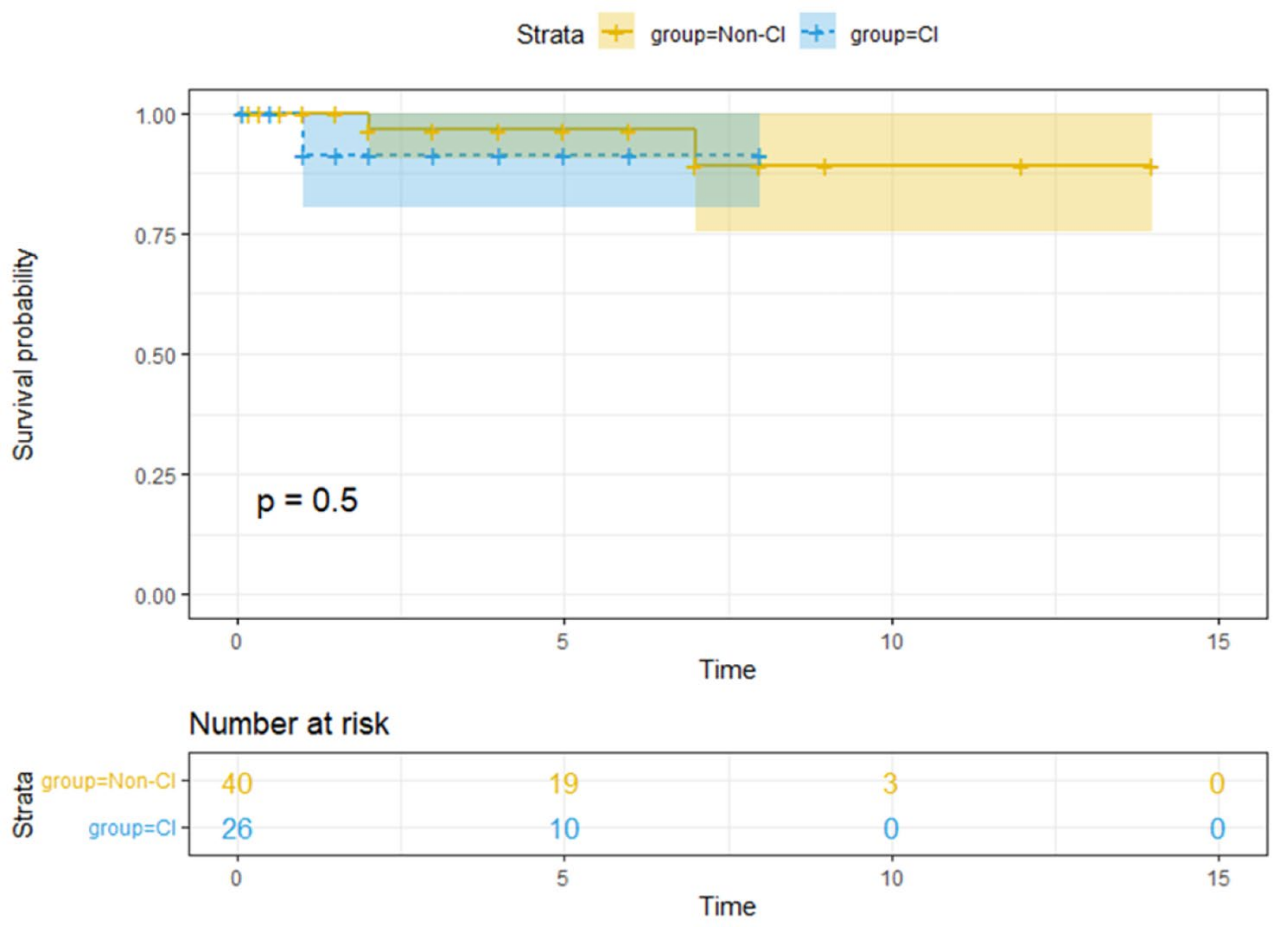
Survival rate analysis of CAL and non-CAL groups after treatment

After treatment, there was no statistically significant difference in the survival rate between the CAL and non-CAL groups (P > 0.05) (Fig. 3).

## Discussion

TAK is a chronic, nonspecific, large- and medium-sized vascular inflammatory disease that mainly affects the aorta and its branches. The coronary artery is an important medium-sized vessel in the heart that may also be involved. Once the lesion is aggravated, thrombosis can develop, leading to MI and even death [23–25]. Even if immediate ischemia does not occur because of coronary artery occlusion, vessels that have been inflamed in systemic vasculitides, such as TAK, show premature atherosclerosis, placing patients at risk for MI [27]. TAK combined with CALs directly affects prognosis ^[28.29]^. Therefore, it is of great significance to screen the risk factors for CAL in TAK patients, and to take appropriate prevention and control measures to improve their prognosis.

Previous studies ^[30.31]^ reported that the incidence of adult TAK combined with CAL varies greatly (3–30%), and the incidence in pediatric TAK patients (55.6%) was significantly higher than in adults [32]. This study shows that pediatric TAK combined with CAL was 39.4%. More attention should be paid to CAL in pediatric patients with TAK. In our cohort, children in the CAL group were all subclinical, the common coronary arteries involved were LMCA and RCA, and some patients may have had four coronary arteries involved. CAL is mostly a small or middle coronary artery aneurysm; some children may have giant coronary aneurysmal dilations, thrombosis, and heart failure. Therefore, once a patient is diagnosed with pediatric TAK, the coronary arteries should be evaluated. Adult TAK usually uses coronary CT or angiography, which is better than coronary ultrasound to identify problems of the distal coronary artery, especially stenosis ^[4.33.34]^. However, we found that the CALs of pediatric TAK were different from those of adult patients, the most common of which was coronary artery stenosis. Coronary ultrasound, which is a noninvasive examination that can detect coronary artery dilation, is more suitable for pediatric TAK coronary artery evaluation. Our results also suggest that pediatric TAK treated with regular glucocorticoids, immunosuppressants, or biological agents was well controlled. Most CALs regressed or returned to normal, and no patient suffered from MI. Two deaths in the CAL group were recorded and considered to be related to irregular treatment. After treatment, there was no difference in the survival rates between the CAL and non-CAL groups. Early detection of CAL and prompt corresponding intervention effectively improved the prognosis of pediatric TAK.

The main mechanism of adult TAK involvement in coronary artery stenosis is inflammation of the aorta extending to the opening and near the segment of the coronary artery, leading to intimal hyperplasia, and fibrosis contracture of the middle and outer membranes causes stenosis of the lumen. Previous studies [10] reported that homocysteine, TG, and the TG/HDL-C ratio were independent risk factors for adult TAK complicated with CAL. Ohigashi et al. [35] reported that the incidence of CAD in adult patients with TAK increased with age. In contrast, Shi-Min et al. [36] reported that the age of patients with TAK combined with CAL was mostly < 40 years. After multivariate analysis, Wang et al. [11] proposed that age at onset, disease course, height, and body mass index (BMI) were risk factors for adult TAK involving the coronary arteries. In summary, inflammation is not the only mechanism in adult TAK patients with CAD related to atherosclerosis. The latter is associated with age, glucocorticoid use, and traditional cardiovascular risk factors. However, in our cohort, these children did not have traditional cardiovascular risk factors and did not receive glucocorticoids. The result suggest that pediatric TAK with CAL is associated with inflammation (higher CRP, WBC, PLT, TNF-α and IL-2R levels, lower HGB ), which differs from that in adults. In addition, our study showed that the course of disease in the CAL group was shorter than that in the non-CAL group. This observation suggests that CAL tended to have a relatively more rapid clinical course and stronger inflammatory state on admission, including destroyed elastic lamina or muscle media and arterial vasodilation. This may partly explain why CALs quickly resolved in children who received biological agents in combination with traditional immunosuppressive therapy.

In our cohort, age at onset was an independent risk factor for pediatric TAK combined with CAL. This indicates that early disease onset in these patients is more likely to cause CAL, especially at less than 36 months. These findings are similar to those reported by Lu, who presented an unusual case of sudden death in an 8-month infant with TAK [37]. An autopsy revealed that the proximal segment of the left and right coronary arteries showed approximately 60% and over 90% occlusion, respectively. It is suggested that we be alert in recognizing and screening CALs on time and pay close attention to the comprehensive control of inflammation, which is very important in improving the prognosis of TAK in children. In addition, this suggests that TAK and KD should be identified in infants with fever and CALs, which was also Reported by Lee et al. [38]. However, Zhao et al. and Filiz Ekici et al. reported some cases of infant KD complicated by systematic artery aneurysms (SAAs) and CAL [39–41]. This makes it more difficult to distinguish between the two types of systemic vasculitis. Taking our patients into consideration, we believe that the following three points will help distinguish between the two diseases. First, no or weak pulse, hypertension, or blood pressure difference between the upper extremities may exist in pediatric patients with TAK. Second, KD has no reports of diffuse aortitis or arterial stenosis, making it distinguishable from TAK. Third, the responses to treatment were different. Patients with IVIG, ASP, and a short course of glucocorticoids that failed to completely control the inflammation were more likely to be pediatric TAK patients.

Lei et al. [12] reported that TAK children with coronary aneurysmal dilation had more aneurysms or dilation of the aorta and its main branches than those with stenotic lesions only. However, no significant differences were observed in the present cohort. We found that pediatric TAK patients with RAS were less likely to develop CAL. It is well known that RAS, which can cause renal vascular hypertension and decrease renal perfusion, is more common in patients with TAK. In severe cases, heart failure and/or renal failure may occur [42]. Renal dysfunction can activate the renin-angiotensin-aldosterone system, cause oxidative stress, and increase the synthesis of endothelin and inflammatory factors,often combined with a variety of risk factors such as old age, obesity, smoking history, hypertension, diabetes, dyslipidemia, and hyperuricemia, which leads to endothelial dysfunction and promotes atherosclerosis [11]. In accordance with this notion, we speculate that adult TAK patients with RAS are more likely to have CALs. However, this is contrary to our results on pediatric TAK. The following points are worth considering: 1.There is little correlation between CAL and atherosclerosis in pediatric TAK. 2. The characteristics of blood vessels in children are related. The arteries in pediatric TAK are in the developmental stage, which are thin wall, low elasticity and shorter diameter than adults. It is more prone to dilation than adults. 3. Is the order of blood vessels affected by vasculitis related to age? Currently, no literature on RAS and CAL has been retrieved. Therefore, further study is needed to investigate the mechanism by which pediatric TAK with RAS are less likely to develop CALs.

This study was limited by its retrospective design, associated biases, and missing data. The relatively small number of samples collected in our single center might have introduced some bias into the results of the comparisons. Nevertheless, our pediatric cohort represents the largest reported worldwide, and we still conclude with significant findings that can guide our clinical work.

## Conclusion

The incidence of pediatric TAK combined with CALs was 39.4%. In the CAL group, 19 (73.1%) cases started within 36 months. None of the patients had symptoms of angina or ischemia on electrocardiogram (ECG), the CALs were detected using coronary ultrasound. The common coronary arteries involved were the LMCA and RCA. CALs are mostly small or middle coronary artery aneurysms, and some children may have giant coronary aneurysmal dilations, thrombosis, and heart failure. This study showed that CAL in pediatric TAK patients has a relatively more rapid clinical course and a stronger inflammatory state at the time of diagnosis. Early onset, especially at less than 36 months, and without RAS is more likely to develop CALs. A thorough assessment of coronary arteries using coronary ultrasound for pediatric TAK without RAS is recommended in the early stage, which is very important in improving the prognosis of pediatric TAK.

## Electronic supplementary material

Below is the link to the electronic supplementary material.

Supplementary Material 1

## Data Availability

All data generated or analyzed during this study are included in this published article.

## List of abbreviations

TAK: Takayasu arteritis
CAL: coronary artery lesion
HGB: Hemoglobin
CRP: C-reactive proteins
WBC: white blood cell
PLT: Platelet
TNF-α: tumor necrosis factor-alpha
IL-2R: interleukin-2 receptor
TG: Triglyceride
HDL-C: high-density cholesterol
AESR: average erythrocyte sedimentation rate
FS: Ferritin
PRE: Prednisone
IL-6: interleukin-6
MRA: magnetic resonance angiography
SD: standard deviation
CT: computed tomography
NIH: National Institutes of Health
LMCA: left main coronary artery
RCA: right coronary artery
LAD: left ascending coronary artery
Cx: circumflex artery
IFX: Infliximab
TCZ: Tocilizumab
CTX: Cyclophosphamide
Y: Year
ASP: Aspirin
p: Patient
m: Month
M: Median
LCA: left coronary artery
FIB: Fibrinogen
CAS: carotid artery stenosis
TAS: thoracic aortic stenosis
AAS: abdominal aortic stenosis
RAS: renal artery stenosis
CI: confidence interval
SAAs: systemic artery aneurysms
MRI: magnetic resonance imaging
KD: Kawasaki disease
IVIG: intravenous immunoglobulin
MTX: Methotrexate
