# Supplementary material for: Clinical characteristics and risk factors of coronary artery lesions in chinese pediatric Takayasu arteritis patients: a retrospective study
Source: Pediatr Rheumatol Online J. 2023 Apr 28;21:42. doi: 10.1186/s12969-023-00820-z (PMC10148487; doi:10.1186/s12969-023-00820-z)
Supplement: Supplementary file 1 — Supplementary Material 1 [file 12969_2023_820_MOESM1_ESM.pdf]

Wiley Editing Services

ENGLISH EDITING CERTIFICATE

This document certifies that the manuscript listed below was edited for proper English language, grammar, punctuation, spelling, and overall style by one or more of the highly qualified native English speaking editors at Wiley Editing Services

Manuscript title

Clinical characteristics and risk factors of coronary artery lesions in Chinese pediatric Takayasu arteritis patients： a retrospective study

Authors

Yingjie Xu

Order No

ZFKMV_2_2

Date Issued

December 12, 2022

This document certifies that the manuscript listed above was edited for proper English language, grammar, punctuation, spelling, and overall style. Neither the research content nor the authors' intentions were altered in any way during the editing process. Documents receiving this certification should be English-ready for publication; however, the author has the ability to accept or reject our suggestions and changes. If you have any questions or concerns about this document or certification, please contact help-cn@wileyeditingservices.com.

Wiley Publishing Services is a service of Wiley Publishing. Wiley's Scientific, Technical, Medical, and Scholarly (STMS) business serves the world's research and scholarly communities, and is the largest publisher for professional and scholarly societies. Wiley is committed to providing high quality services for researchers. To find out more about Wiley Editing Services, visit http://wileyeditingservices.com. To learn more about our other author services provided by Wiley

WILEY

Publishing, visit https://authorservices.wiley.com/
